# 

**Published:** 1891-02-21

**Authors:** 


### NOTICE TO CORRESPONDENTS.

All MS., Letters, Books for Review, and other matters intended for the Editor should be addressed The Editor, The Lodge, Porchester Square, London, W.

The Editor cannot undertake to return rejected M.S., even when accompanied by stamped directed envelope.

### Notice to Advertisers and Subscribers.

All Advertisements, Orders for Copies of The Hospital, and Business Communications should be addressed to The Publisher, *not to the Editor*, The Hospital, 140, Strand, W.C.

The Cost of Subscription, post free, is as follows:—Per week, 1½d.; per quarter, 1s. 8d.; per half-year, 3s. 3d.; per annum, 6s. 6d. Foreign:—Per annum, 8s. 8d.; payable, in all cases, in advance.

### GENERAL CHARITIES.

[This Column is devoted to an account of work of charitable institutions other than Medical Charities. The Editor will be glad to receive reports or news of work at 140, Strand. *Philanthropy* should be plainly written on the envelope.]

One of the oldest and most useful of the institutions for orphans in this country is the **ORPHAN WORKING SCHOOL**, which was established as far back as 1758. It can, therefore, boast a respectable antiquity, and what is more to the point, of a successful and highly useful career. The first school was at Hoxton, where a beginning was made with twenty boys, and such was the success of the pioneers, and so rapidly did funds come in that very soon afterwards twenty girls were admitted to the benefits of the charity. How the numerous and competing charities of the present day would like to experience the novel sensation of funds galloping into their coffers instead of crawling in and galloping out! In the early days of the institution, industrial training rather than general education of the children seems to have been the main object of the founders, and their time was chiefly occupied in making shoes and garden nets. How little attention was paid to education is evidenced by the fact that it was not till six years after the foundation of the charity that the simplest form of arithmetic was taught. Yet, notwithstanding this early deficiency, the training since given has produced men who have held honourable positions in society. In these early days also the dress of the children had the stamp of charity marked upon it in a distinctive manner which has since been wisely withdrawn. After a lapse of fifteen years, a second and larger school, to contain seventy children, was erected in the City Road, but this again in course of time became too small, and in 1847 the school was removed to its present site at Haverstock Hill, and enlarged in 1860. A liberal education is now given to the children, who are divided into senior and junior branches, the latter being situated at Hornsey Rise, and the number of children reaping the advantages of the institution is nearly seven hundred. The children, who are admitted between infancy and the age of eleven, remain at school till they are fourteen years of age, and all after being placed out in the world are kept in sight by the management for a period of seven years. The elections take place half-yearly, in January and July, every candidate being nominated by a Governor. Children in delicate health or suffering from any infectious disease or mental or physical defect are not admitted. In addition to the ordinary branches of education, extra subjects are taught before and after ordinary school hours, which have enabled many pupils to distinguish themselves in the Science and Art Examinations at South Kensington. Both boys and girls are drilled and taught to swim, and the girls are taught to cook, as well as to sew, and to be competent to fill any position in life to which they may be called. An excellent feature in the institution is the convalescent home at Margate, where those who fall ill can get the much-needed change and recover their health and strength. Donations and annual subscriptions will be gratefully acknowledged by the Secretary, Mr. Jonadab Finch, 73, Cheapside, E.C.

It is now just fifty years since **THE FIELD LANE INSTITUTION** began its operations in a small room in Caroline Court, Saffron Hill, amidst much ridicule and no small opposition. Ridicule has long since hidden its diminished head, while the Institution has annually enlarged its scope. Its work lay chiefly among the numerous pickpockets in the neighbourhood. Five children were first taken in hand, but the numbers rapidly increased and before long the benefits of the Institution were extended to grown up men and women of the class who are to be found nightly sleeping in the streets, and a perfect network of charitable operations has since been formed. The Society deals with the poorest classes who live in the depths of human misery, on whom it seeks to confer immediate and lasting benefits. The male and female refuges are open all the year round for homeless and destitute people, who are admitted after being questioned by an experienced superintendent, but strict inquiries are subsequently and immediately made. Thus applicants for admission are saved from starving or dying whilst time is being taken up in the inquiries. A record is kept, so far as is possible, of the careers of those who have been in the refuges, and the result is often eminently gratifying to the managers and supporters of the charity. Perhaps the branch which is best known is the ragged school, which is the centre from which all the other branches have since radiated. Connected with it is the day nursery, which has proved an immense boon to poor mothers who have to assist in gaining their own living as well as looking after their husbands. The feature of the institution is the parental system, under which the children, instead of being turned out into the street untaught and uncared for are properly looked after. Infants are taken charge of during the day in the crèche, and the school is attended by many thousands of children in the course of the year. In connection with it there is a lending library which circulates books of an interesting and instructive character. In addition to the ragged school there are also industrial schools for boys and girls. At the close of their terms the children are either transferred to other schools or placed in employment. Not the least useful branch of the Institution is the Servants' Training Home, where an endeavour is made to transform girls from vagrant street hawkers into respectable domestic servants, thereby conferring a substantial benefit not only on the girls, but on society at large. Out of twenty-six girls who, during the last financial year were thus removed from contaminating surroundings and placed under wholesome influences, fourteen were sent out into domestic situations. Those who regularly carry on a system of home visitation on behalf of the Institution see too clearly the misery that exists in many of the dwellings of the working classes notwithstanding all the efforts, legislative and otherwise, which have been made to improve them, and that this misery is not always the result of drink and idleness, but sometimes of enforced struggling against the inability to get decently remunerative employment. In its endeavours to lift up the fallen and to rescue the perishing, the Institution has done much good work, and work of an eminently useful nature. Without the influences which it brings to bear there would be many more worthless and dangerous members in society's midst, and society cannot show in any more practical form its gratitude for further favours to come than by sending donations, in support of a work which rids it of many evils, to the Secretary, Mr. Peregrine Platt, Vine Street, Clerkenwell Road, E.C.

### SCRAPS AND GLEANINGS.

DR. PRADERE has invented a new treatment for tuberculosis.

DR. KOCH'S lymph has received the name of Tuberculine in medical circles in Berlin.

DURING 1890 4,622 patents attended at the Glasgow Dental Hospital; of these 3,391 were cases of extractions.

THE seventh International Congress of Hygiene and Demography will be held in London from August 10th to 17th.

MR. C. A. MORTON, F.R.C.S. (Eng.), has been appointed pathologist to the Bristol Hospital for Sick Children and Women.

A PORTION of St. Mary's Hospital, Rochester, U.S.A., has been burned down. 250 patients and 19 nurses were removed in safety.

COLONEL SEELY has contributed £10,000 towards the establishment of the proposed Nottingham and Notts Convalescent Homes.

THE collections in the churches and chapels of Hastings on Hospital Sunday (January 25th) amounted together to £411 19s. 9d.

DR LEET publishes a letter on ship sanitation, in which he complains that the regulations with regard to ventilation are not complied with.

A NEW Hospital for Women in Glasgow was formally opened by the Countess of Aberdeen on January 30th. There is accommodation for ten patients.

THE fourth annual report of the Beverley Dispensary and Hospital states that during 1890 79 in-patients were under treatment, and 1,161 out-patients.

THE project of a gigantic Koch Hospital, to be erected near Berlin, has been abandoned. Three barracks, with accommodation for 120 patients, will be erected.

THE income of the Helensburgh Infirmary was not sufficient to meet the expenditure. One hundred and fifty pounds was withdrawn from the capital account to make up the deficiency.

THE annual report of the National Children's Hospital, Dublin, states that during the year three new cots have been founded. The debt on the hospital has been reduced from £500 to £300.

THE second annual report of the Cottage Hospital at East Grinstead is satisfactory in every respect. Forty-seven patients received treatment, and the income was more than sufficient to meet the expenditure.

THE managers of the East London Hospital for Children have issued a pretty little appeal for funds, which are urgently needed to carry on the work. It is intended that the hospital should be practically free.

THE Committee of Management of the Bristol General Hospital appeal to the public for a sum of £20,000, which is to be invested, and the interest to be used to meet the extraordinary expenses of the institution.

A GRAND bazaar and fancy fair has been given in Cheltenham on behalf of the Children's Hospital. The promoters, who put forth great efforts to make it successful, have every reason to feel satisfied with the result.

THE discovery by Professor Virchow that certain poisons are contained in Dr. Koch's lymph which produce necrosis, has led Dr. Weyl to discover a means of detecting these poisons and isolating them from the lymph.

A NEW hospital for Egyptian natives has just been opened at Luxor. There are forty beds, all free. The doctor, who is an Egyptian gentleman, was trained at St. Thomas's Hospital, London. Over fifty natives attended for advice on the opening day.

DR. LAYLEY'S report of the work at the London Fever Hospital for last year states that the majority of the patients were clerks, employés domestic servants, and their families; 616 patients were admitted; the rate of mortality was about 3·2 per cent.

UNDER the presidency of the Lord Mayor, the fifty-fifth annual meeting of the subscribers to the St. Mark's Hospital, City Road, was held on the 12th inst. at the Mansion House. The report stated that during the year 310 in-patients and more than 2,000 out-patients had been treated.

IN the New England Hospital for Women and Children, Boston, the physicians and surgeons are all women. The report for 1890 shows that the hospital has progressed with unabated zeal and proportionate success. The total number of patients admitted was 466; 147 operations were performed, of which almost all were successful.

PRINCE CHRISTIAN presided at the annual meeting of the Governors of the Windsor Royal Infirmary, on January 30th. Mr. F. T. Barry, M.P., in moving, and the Dean of Windsor in seconding, a vote of thanks to his Royal Highness, made earnest appeals on behalf of Princess Christian's Nurses' Fund, which, it was urged, was doing much good in the town and district.

### The Student of Medicine.

[All communications for this Supplement should be addressed to The Editor of The Hospital, at 140, Strand, with the words, "Students' Column," written in the left hand top corner of the envelope.]

### APPOINTMENTS.

At the Chelsea Hospital for Women, A. D. Leith Napier, M.D., F.R.S.Ed., M.R.C.P., has been appointed physician to out-patients. At the New Hospital for Women, Euston Road, Ellen M. T. Berththon, M.B.Lond., has been appointed clinical assistant. At the Royal Orthopædic Hospital, Johnston Macgregor, M.R.C.P., L.R.C.P., has been appointed resident house surgeon and apothecary. At the Poplar Hospital for Accidents Ernest James Reynolds, M.R.C.S., L.R.C.P., has been appointed senior house surgeon. At St. Thomas's Hospital, Felix Semon, M.D.Berlin, F.R.C.P.Lond., has been appointed physician for diseases of the throat. At the Farringdon General Dispensary and Lying-in Charity, James Crawford, M.D., M.R.C.P.Lond. and Irel.L.M.,as been appointed honorary physician-accoucheur. At the Devon and Exeter Hospital, Henry Andrew, M.R.C.S., L.R.C.P., has been appointed assistant house surgeon. At the General Hospital, Birmingham, H. A. Berryman, M.R.C.S., L.R.C.P., has been appointed assistant house surgeon. At the Ipswich and East Suffolk Hospital, H. C. Critchley, M.B., C.M., L.R.C.S.Edin., has been appointed senior house surgeon. At the Manchester Hospital for Consumption, William Milligan, M.B., C.M., has been appointed honorary assistant physician. At the Warwick Dispensary and Cottage Hospital, Robert F. T. Perkin, L.R.C.P.Edin., M.R.C.S., has been reappointed visiting surgeon. At the Torbay Hospital, Reginald Pollard, M.B., M.R.C.S., has been appointed honorary surgeon. At the City Hospital for Infectious Diseases, Newcastle-on-Tyne, B. T. Stokoe, M.B., B.S., has been appointed resident medical assistant. At the Manchester Southern Hospital, and the Manchester Maternity Hospital, A. W. Senior, M.R.C.S.Eng., L.R.C.P.Lond., has been appointed house surgeon.

### VACANCIES.

The following vacancies are announced this week, the amount of salary in each case being given after the name of the institution:

### Resident Medical Officers.

Addenbrooke's Hospital, Cambridge, House Physician—Salary £65.
Brixton, Streatham, and Herne Hill Dispensary, Resident House Surgeon—Salary £150, &c.
Central London Sick Asylum District, Assistant Medical Officer and Dispenser—Salary £100, &c.
Chichester Infirmary, Chichester, House Surgeon—Salary £80, &c.
City Hospital for Infectious Diseases, Newcastle-on-Tyne, Resident Medical Assistant—Salary, £50, &c.
Cheltenham General Hospital, Junior House Surgeon—Salary £40, &c.
Doncaster General Infirmary and Dispensary, House Surgeon—Salary £100, &c.
County Donegal Infirmary, House Surgeon and Registrar—Salary £60, &c.
General Hospital, Birmingham, Resident Medical Officer—Salary £130, &c.
Great Northern Central Hospital, Holloway Road, N., Casualty Officer to act as Registrar and Anæsthetist—Honorarium 50 guineas.
Hospital for Consumption and Diseases of the Chest, Brompton, House Physicians—Board, &c.
Hospital for Sick Children, Great Ormond Street, Bloomsbury, Surgical Registrar for one year—Honorarium £40.
Liverpool Royal Southern Hospital, Junior House Surgeon—Salary 60 guineas, &c.
National Dental Hospital, Great Portland Street, W., House Surgeon—Salary £50.
Newcastle-on-Tyne Dispensary, Resident Medical Officer—Salary £250, &c.
North Staffordshire Infirmary and Eye Hospital, Hartshill, Stoke-upon-Trent, Assistant House Surgeon.
Nottingham General Hospital, Resident Medical Assistant and Resident Surgical Assistant—Board, &c.
Royal Hospital for Sick Children, Glasgow, Assistant House Surgeon—Salary £30, &c.
Royal Victoria Hospital, Bournemouth, House Surgeon and Secretary—Salary £30, &c.
Salford Union, Two Resident Assistant Medical Officers—Salary £130, &c., each.
Scarborough Hospital and Dispensary, House Surgeon—Salary £80, &c.

### The Hospital Advertiser.

**THE HOSPITAL FOR SICK CHILDREN,**
GREAT ORMOND STREET, BLOOMSBURY, W.C.

### THE STUDENT OF MEDICINE—(*continued*).

Southern Hospital, Clifford Street, Manchester, House Surgeon.
West Bromwich District Hospital, Assistant House Surgeon.

### Non-Resident Medical Officers.

Brown Animal Sanatory Institution, Professor Superintendent—Salary £250.
St. George's and St. James's Dispensary. Surgeon.
Bristol Hospital for Sick Children and Women, Medical Registrar.
Cambridge, Assistant Medical Officer for the Cambridge Friendly Societies' Medical Association.
Great Northern Central Hospital, Holloway Road, Surgeon to the Out-patients.
Grosvenor Hospital for Women and Children, Westminster, S.W., Surgeon.
Owens College, Manchester, Demonstrator in Pathology—Stipend £140.
Royal South London Dispensary, Surgeon-in-Ordinary.
Royal Academy of Arts, Professor of Anatomy.
Salford Union, a Visiting Medical Officer—Salary £150.
St. Thomas's Hospital, Westminster, Surgeon.
University of Glasgow, Examiners in Medicine—Annual fee £30 each.

### PASS LISTS.

### Society of Apothecaries of London.

The following have passed the first examination in chemistry, materia medica, botany, and pharmacy: D. Berne, Sydney University; R. B. Greaves, Sheffield; E. Grange, Edinburgh; T. L. Johnston, Edinburgh University and University College.

The following passed in the subjects indicated:—Materia medica, botany, and pharmacy—E. Dodds, Sheffield, and T. H. P. Peers, Charing Cross Hospital; materia medica and botany—K. M. Hunter, Royal Free Hospital, and F. K. Rider, Leeds Yorkshire College; pharmacy—F. R. Rouse, University College.

The following passed the second examination in anatomy and physiology:—W. Ashby, Guy's Hospital; G. H. Baird, Dublin Carmichael College; G. W. Brabyn, St. Mary's Hospital; E. S. Chilcott, St. Mary's Hospital: S. A. Clarke, Cork Queen's College and Middlesex Hospital; T. S Collin, Victoria University, Owens College: F. A. Cregeen, Cambridge and Liverpool University College; A. B. Franey, St. Mary's Hospital; A. H. Grace, Bristol; G. Higginson, Cambridge and London Hospital; H. Howells, St. Thomas's Hospital; T. S. F. Hudson, Birmingham, Queen's College; W. A. King, Charing Cross Hospital; A, E. Marwood, Charing Cross Hospital; S. R. Merry, Charing Cross Hospital; J. C. Netherton Dublin Trinity College; C. St. H. Robinson, St. Mary's Hospital; J. D. Seller, St. Mary's Hospital; J. R. Shotton, Leeds, Yorkshire College; J. Stott, Oxford and Victoria University, Owens College; R. W. F. Welch, St. Thomas's Hospital.

The following passed in anatomy: C. Bayley, Edinburgh University; G. E. Douglas, St. Mary's Hospital.

The following passed in physiology: A. Baldwin, Birmingham, Queen's College; H. C. Venis, Calcutta and St. Mary's Hospital.

The following candidates passed in surgery: T. H. Adams, London Hospital; W. L. W. Buss, Cambridge and Middlesex Hospital; R. A. Earle, Middlesex Hospital; E. J. Finch, St. Mary's Hospital; R. A. Irvine, Belfast, Queen's College, and Glasgow; A. E. Mayner, M.D., Bishop's College, Montreal; B. F. Parish, St. Mary's Hospital; G. J. Rutherford, Middlesex Hospital; S. C. Smith, Middlesex Hospital; J. D. H. Smyth, Belfast, Queen's College, and St. Mary's Hospital; G. R. F. Stilwell, St. Thomas's Hospital; J. F. Twist, Birmingham, Queen's College; E. B. Wrench, Cambridge and St. Thomas's Hospital.

The following candidates passed in medicine, forensic medicine, and midwifery: C. A. Beck, Bonn University and Middlesex Hospital; L. J. Minter, King's College; R. B. Morris, Manchester, Owens College: G. Wilkinson, Cambridge and Middlesex Hospital.

The following passed in medicine and midwifery: G. C. W. Williams, St. Thomas's Hospital; E. H. Willcock, St. Thomas's Hospital.

The following passed in medicine: P. G. Laver, St. Thomas's Hospital.

The following passed in forensic medicine: H. C. Coopland, St. Bartholomew's Hospital.

To Messrs. Adams, Beck, Buss, Finch, Irvine, Morris, and Wilkinson were granted the diploma of the society, having passed in all the subjects required for registration.

### Royal College of Physicians of London.

The following gentlemen having conformed to the bye-laws and regulations, and passed the required examinations, were, at a meeting of the College on January 29th, admitted Licentiates: W. Bragg Addison, St. Bartholomew's and Cambridge; *M. Adler, Guy's and St. George's; J. E. Felix André, St. Thomas's; Reginald F. E. Austin, Bristol; A. Keppel Barrett, St. Mary's; F. Warburton Begbie, St. Bartholomew's; C. J. Blakeman, St. Thomas's; H. F. S. Blake, Charing Cross; C. Stanser Bowker, Middlesex; *E. T. A. Boyton, St. Bartholomew's; *W. Charles Burt, Guy's; W. F. Chambers, University College; *G. W. Chapman, St. Thomas's; A. Reginald Chater, St. Mary's; W. Adams Clark, St. Bartholomew's; Hugh Clift, St. Bartholomew's; Thomas Sandby Coombe, Guy's; C. Arthur Coventon, St. Bartholomew's; A. Rodgers Cowell, St. Thomas's; Roger Crosskey, St. Thomas's; H. Jones Curtis, University College; W. P. Taylour Daniel, St. Mary's; Matthew Dobbs, Charing Cross; J. Hardman Dow, Manchester; E. Charles Drake, St. Bartholomew's; L. Winter Dryland, St. Bartholomew's; *S. Unwin Duer, Middlesex; P. Joseph Duffy, University College; Percy Evans, University College; W. G. Robertson Farquharson, St. Mary's; Frederick Arthur Field, St. Bartholomew's; T. A. Munro Forde, St. Thomas's; *Francis Wheldale Foster, Guy's; Reginald Stilwell Freeland, Guy's; L. Parker Gamgee, Birmingham; Harry Gordon, Manchester; Michael Grabham, St. Thomas's; J. H. P. Graham, St. Bartholomew's;

### The Hospital Advertiser.

INVALIDS' Furniture, BATH CHAIRS & PERAMBULATORS
ALFRED CARTER, 47, Holborn Viaduct, E.C.

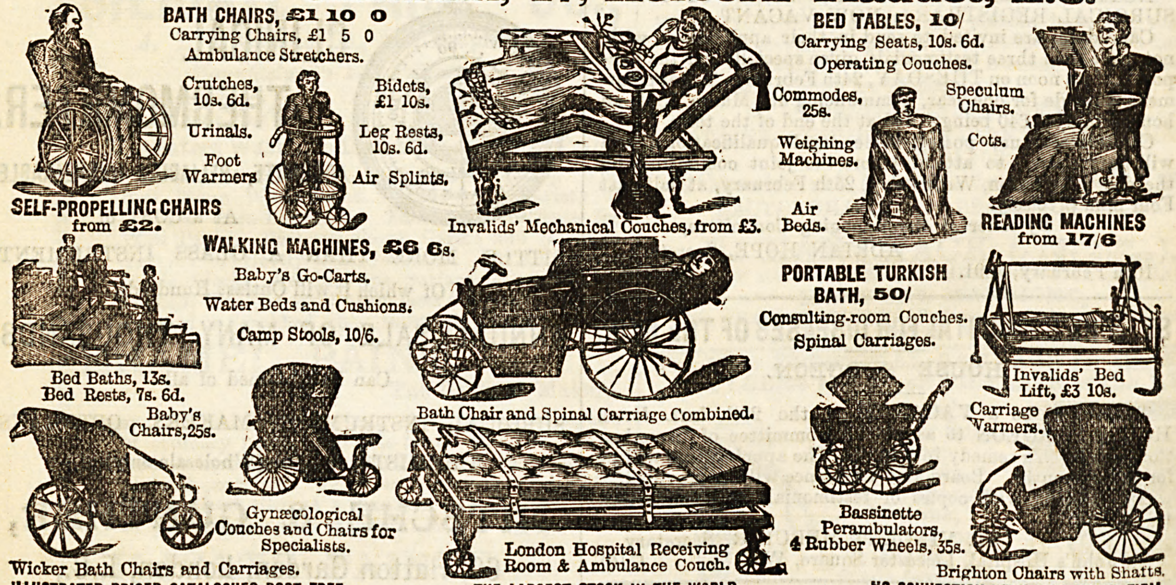

ILLUSTRATED PRICED CATALOGUES POST FREE. THE LARGEST STOCK IN THE WORLD. NO CONNECTION WITH ANY OTHER FIRM.
ALFRED CARTER, 47, Holborn Viaduct, E.C.

Keen's Mustard

THE OLDEST MANUFACTORY IN THE CITY OF LONDON.

BRINSMEAD PIANOS

### THE STUDENT OF MEDICINE—(*continued*).

C. Albert Green, Birmingham; Percy Andrew Green, London; R. E. Greenwood, University College; Gilbert H. Griffiths, Liverpool; *John Samuel, Griffiths, Bristol; John Grimshaw, London; Walter George Gyton, Manchester; *Samuel Hawarden, Manchester; *Frank Haydon, Westminster; T. Horatio Haydon, St. Thomas's and Cambridge; Charles Harold Hemming, London; John George Hewitson, University College; Frederick Robert Hird, Leeds; *E. Burke Holland, Middlesex; *A. Walters Howard, University College; Thomas H. Kellock, St. Thomas's; Archibald Kidd, Middlesex; T. A. O. Langston, St. Bartholomew's; H. Driffield Levick, St. Thomas's: Frederick Lewis, St. Mary's; C. Petre Lovell, St. Thomas's; Vincent Warren Low, St. Mary's; J. W. Mactavish, St. Thomas's; E. E. Manwaring, St. Bartholomew's; Nicholas Marder, St. Bartholomew's; E. W. G. Masterman, St Bartholomew's; John B. Mayor, Manchester; Yarnold Hubert Mills, London; *John More, St. Bartholomew's; Edwin Haigh Grant Morris, Cambridge and St. Thomas's; J. J. N. Morris, King's College; Thomas E. Mulvany, London; Robert Henry Norgate, Bristol; Arthur J. Nyulasy, Melbourne; S. A. Ord-Mackenzie, University College; Herbert Lloyd Penny, London; George Pernet, University College; T. M. J. Powell, St. Bartholomew's; E. L. Pritchard, King's College; *Edward Melville Quinby, Liverpool and St. Bartholomew's; H. Reeks, St. Bartholomew's; A. E. Reynolds, University College; Leonard Rogers, St. Mary's; J. M. Rogers-Tillstone, St. Bartholomew's; Robert Miller Ronaldson, Edinburgh and Charing Cross; John C. Round, St. Thomas's; R. G. Rows, University College; Henry Bertram Rygate, Guy's; Daniel West Samways, Guy's; Arthur W. Senior, Manchester; R. W. Senior, King's College; *E. D. Shirtliff, St. Thomas's; *Henry Smith, King's College; Charles E. Saulby, Middlesex; James Stalker, Leeds; R. Stephens, St. Bartholomew's; Alfred William Sturdee, London; William H. Sturge, London; Thomas C. Summers, London; Arthur Hugh Thompson, Cambridge and London; *Walter Twyford, Manchester; E. J. C. Tyler, St. Thomas's; *John Alfred Ward, Guy's; J. A. Waring, University College; *Stawell Weldon, St. George's; Arthur Whitfield, King's College; J. P. Wightman, St. Bartholomew's; J. H. Wilks, St. Bartholomew's; T. Willey, St. Bartholomew's; David J. Williams, Middlesex; Walter Winslow, Guy's; Cuthbert Wyman, St. Thomas's and Cambridge; John Young, Guy's.

*Candidates who have not presented themselves under the regulations of the Examining Board.

### ATHLETICS.

### Football.

HOSPITAL CUP.—On Monday, 2nd, *Guy's* played *London*. The game throughout was very one-sided, and much disappointed the expectations of the large number of spectators present. London, who kicked off, never once threatened danger. Directly after the start, Jewitt obtained a try for Guy's, which Mitchell failed to convert. Shortly afterwards, however, Mitchell dropped a splendid goal. Guy's still kept scoring, and before half-time arrived Bettington, Cooper, and Swayne all got behind. In each instance Mitchell sent the ball between the uprights. At half-time the score was three goals and two tries to nil in favour of Guy's. In the second half the London men played up better, and for some time no score was added by either side. At last Cooper, by a very good run, grounded the ball behind the uprights, and Mitchell again converted it into a goal. Then London pressed, and a few minutes before call a try by Lewis was converted by Rigby, Guy's thus winning by four goals and two tries to one goal, or thirteen points to three. Referee, Mr. F. W. J. Goodhue (Thomas's). The draw for the second round is as follows: February 10th, Charing Cross v. Guy's; February 12th, St. Thomas's v. St. George's; Middlesex (a bye). The semi-final will be played on February 24th, and the final on March 5th. The rules of scoring have been altered to the following: A try, or penalty goal, one point; a goal from the field of play, two points; a goal from a try, three points.

ASSOCIATION RULES.—FEBRUARY 7.

*Cambridge University* beat *Casuals* at Cambridge by nine goals to nil. *University College Hospital* was beaten by *Clapham Rovers* by four goals to nil. *St. George's Hospital* was beaten by *Clapham Rovers*, at Wandsworth, by two goals to one. *Oxford University* beat *Old Westminsters*, at Oxford, by seven goals to two. *St. Bartholomew's Hospital* was beaten by *Royal Arsenal* at Plumstead, by five goals to four.

RUGBY.—FEBRUARY 7TH.

*Guy's Hospital* was beaten by *Blackheath*, at Blackheath, by six goals and three tries to nil. *Cambridge University* beat *Richmond*, at Richmond, by three goals to two tries. *London Hospital* beat *Wasps*, at Putney, by two goals and one try to nil. *Middlesex Hospital* beat *Brighton*, at Brighton, by one goal and one try to one try. *Oxford University* beat *Harlequins*, at Oxford, by five tries to nil. *St. Thomas's Hospital* beat *Clapham Rovers*, at Wandsworth, by one goal and one try to nil.

### Edinburgh University Hare and Hounds Club.

January 24th, from Duddington, eight miles. January 31st, from Currie, seven miles, in very bad weather.

### EVENTS FOR THE MONTH OF FEBRUARY.

[Items intended for this column must reach the office, 140, Strand, W.C., not later than first post on Monday mornings.]

For list of Students' Medical Societies' Meetings and Football Matches during February, see THE HOSPITAL for Feb. 7th.

